# A Rare Case of a Spontaneous Thoracic Epidural Hematoma in a Young Male Weightlifter

**DOI:** 10.7759/cureus.59889

**Published:** 2024-05-08

**Authors:** Max Kabolowsky, Kaitlyn Pearl, Meilani Mapa, Ariel Inocentes

**Affiliations:** 1 Medicine, Broward Health North, Deerfield Beach, USA; 2 Dr. Kiran C. Patel College of Osteopathic Medicine, Nova Southeastern University, Fort Lauderdale, USA; 3 Physical Medicine and Rehabilitation, Broward Health North, Deerfield Beach, USA

**Keywords:** extradural mass, weight lifting, american spinal injury association impairment scale, neurologic impairement, hemilaminectomy, spontaneous spinal epidural hematoma (sseh)

## Abstract

Spontaneous spinal epidural hematoma (SSEH) is the accumulation of blood in the epidural space of the spinal cord. Acute SSEH is a rare phenomenon that presents with a wide variety of neurologic symptoms and most often is a surgical emergency. We present a previously healthy 34-year-old male with sudden onset progressive weakness and tingling in the right lower extremity that progressed to the left lower extremity while bench pressing weights, resulting in complete lower extremity paralysis. Magnetic resonance imaging (MRI) revealed a 3.0 cm extradural mass centered in the dorsal and left lateral canal. After a T1-T4 hemilaminectomy was performed which was followed by inpatient rehabilitation, the patient had a favorable outcome improving from The American Spinal Injury Association Impairment Scale (AIS) grade A, complete impairment, to AIS grade C, incomplete impairment on discharge. Initially, the patient had complete motor and sensory paralysis below the level of T4, and upon discharge, the patient was able to attain modified independence in activities of daily living, mobility, and transfer. Due to the lack of risk factors for SSEH in this patient, the etiology is most likely related to the Valsalva maneuver while weightlifting. Lesions in the thoracic region with rapid progression of neurologic symptoms are indicators of poor prognosis, so this case highlights the importance of prompt recognition and intervention for improved outcomes to prevent devastating neurologic defects.

## Introduction

Spontaneous spinal epidural hematoma (SSEH) is a rare clinical manifestation occurring from the blood accumulating in the epidural space, resulting in spinal cord compression and neurological deficits most commonly without known trauma or other iatrogenic causes. SSEH affects mostly the thoracic region in the fourth or fifth decade of life with an estimated incidence of 0.1 patients per 100,000 annually [1,2]. Lesions can occur anywhere along the spinal tract; however, lesions in C6 to C7 and T12 are more common. The etiology of SSEH is largely unknown, but predisposing factors include bleeding disorders, anticoagulants, antiplatelets, thrombolytics, pregnancy, and vascular malformations [3]. Factors that may suggest a poor outcome on presentation include a hematoma in the thoracic region, the use of anticoagulants, a sphincter dysfunction, and a rapid progression [4]. Conversely, factors that may suggest a better prognosis include incomplete neurologic deficits and early surgical intervention. Surgical intervention of decompressive laminectomy is often warranted given the risk of permanent neurologic sequelae. Conservative management is a treatment option in those that are not ideal surgical candidates, often secondary to bleeding risks [3]. To standardize spinal cord injuries, guide further assessment and treatment, and classify injuries as either complete or incomplete which has an important neurologic prognostic outcome, The American Spinal Injury Association Impairment Scale (AIS) is used [5]. The scale ranges from complete impairment, AIS-A, to normal function, AIS-E.

Building on this background, we present a case that illustrates the critical nature of timely intervention in SSEH. We describe a case of a SSEH occurring in the thoracic region after lifting weights. The initial grade was AIS-A; however, after surgical intervention and physical therapy, the patient was discharged as AIS-C. This case emphasizes the importance of early recognition and surgical treatment of this rare pathology to improve outcomes and decrease debilitating deficits.

## Case presentation

**Figure 1:**
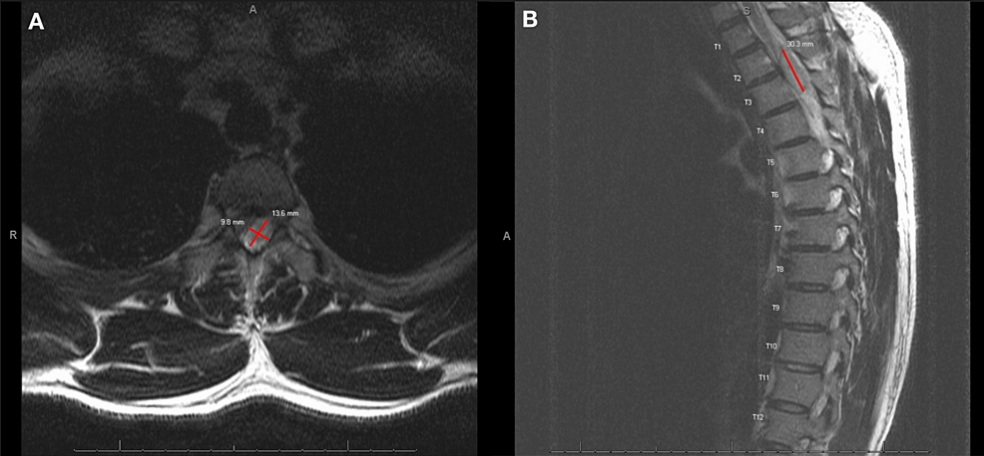
(A) Preoperative hypertense 3.0 cm extradural mass centered in the dorsal and left lateral canal at T2 on T2-weighted imaging on axial view highlighted in red. (B) Preoperative hyperintense 3.0 cm extradural mass centered in the dorsal and left lateral canal at T2 on T2-weighted imaging on sagittal view highlighted in red

A 34-year-old male with no significant past medical history presented to the emergency department with weakness of the bilateral lower extremities. While bench pressing 90 lbs, he had sudden weakness and tingling of the right leg, and upon standing from the bench, the patient’s weakness quickly progressed to the opposite leg. Subsequently, the patient lost all sensation and movement in his lower extremities and was brought to the emergency room. On the initial physical exam, the patient had normal tone and strength in the bilateral upper extremities with intact sensation between C1 and T2. He reported partial sensation in the T3 dermatome, but absent sensation of the T4 distribution and below. At the lower spine, he had an inability to feel sharp sensation; however, he was able to feel the pressure in the lower extremities. There was an increase in the tone of the lower extremities, absent motor function below the T4 distribution, and no evidence for clonus. Bilateral lower extremity compartments were soft and compressible with 2+ dorsalis pedis pulse. Magnetic resonance imaging (MRI) of the thoracic spine revealed a 3.0 cm extradural mass centered in the dorsal and left lateral canal at T2 (Figure 1) resulting in spinal cord compression.

The patient was taken to the operating room and underwent a left-sided T1-T4 hemilaminectomy for epidural hematoma evacuation. Six days postoperatively, spinal angiogram showed no evidence of dural arteriovenous (AV) fistula in the spinal cord. The following day, the patient was admitted to inpatient rehabilitation and began bedside physical and occupational therapy which continued over the next three weeks. Upon discharge, the patient was able to attain modified independence in activities of daily living, mobility, and transfer. He required maximum assistance in the sit-to-stand test and was unable to ambulate due to paraplegia [6]. Patients' discharge rectal exam showed firm rectal tone with excellent squeeze effort as well as reported full sensation of the exam.

On initial presentation, the patient was graded as AIS-A, with complete impairment and no motor or sensory function below the level of the injury. After hemilaminectomy and three weeks of occupational and physical therapy, the patient was discharged as AIS-C with incomplete impairment and preservation of some motor function below the neurological level but with more than half of the key muscles below the level having a muscle strength less than three, unable to move against gravity [7]. 

Seven months postoperatively, the patient continues in skilled physical therapy, focusing on wheelchair mobility with ramps and curbs to improve independence. Other goals include focusing on standing and pre-gait activities; however, the patient has increased tone in bilateral lower extremities during standing activities requiring an increase in the muscle relaxant, baclofen.

## Discussion

Spontaneous spinal epidural hematomas (SSEHs) cause a variety of debilitating neurologic deficits and may even result in death. SSEH commonly presents with intense back or neck pain with symptoms ranging from nerve root damage to complete loss of neurological function. The diagnosis of SSEH is often difficult unless significant neurological symptoms arise. This often leads to a delay in diagnosis which is attributed to poor long-term outcomes. Thus, a high degree of suspicion, and a subsequent quick diagnosis, can allow for immediate surgical interventions if warranted to prevent permanent neurologic damage [1]. 

MRI stands as the imaging modality of choice to diagnose SSEH, as it is easily accessible in emergency room settings, and has enhanced contrast resolution allowing for a heightened sensitivity in detecting acute soft tissue and spinal cord injuries [1,8]. CT is a lesser alternative when MRI is not available [1]. Acute SSEHsare most common and are often related to trauma, hematologic disorders, and medications [3]. Chronic SSEHs are less common with most being idiopathic [9]. On imaging, 24 hours after hematoma formation in acute SSEH, the hematoma usually presents as hyperintense on T2-weighted MRI and hypointense on T1-weighted MRI imaging, whereas chronic hematomas present as hypointense on both T1 and T2-weighted imaging [10-12]. Therefore, a hyperintense hematoma on T2 imaging in our patient with an acute SSEH further solidified the diagnosis and guided decision-making on further treatment. 

Surgical intervention for SSEH is most commonly a hemilaminectomy or laminectomy. Due to the absence of motor and sensory function below T4 seen in the patient we present here, urgent decompressive surgery was indicated to prevent permanent paralysis. When surgical intervention is not an option, or the risks outweigh the benefits, conservative management of strict bedrest with continued monitoring and serial examinations is the best course of action [1,13]. Patients with incomplete neurological deficits, lesions not in the cervical region, and lesions less than four vertebral segments have a better prognosis [10]. 

Due to the rarity of this condition, risk factors are not well established; however, there have been associations between sex, age, hypertension, and vascular malformations. This disease process seems to favor men and individuals past their fourth decade of life [14]. Certain blood dyscrasias, disorders of the blood, and bone marrow clotting proteins or lymph tissue have also been suspected to play a role in the development of SSEH [15]. Tawk et. al discussed a case of a 49-year-old male with a factor V Leiden mutation that presented with progressive motor deficits that led to total paralysis [13]. Other possible predisposing factors, such as arteriovenous malformations have also been described. Huang et. al presented a unique case of a patient with undiagnosed arteriovenous malformations that had undergone spinal manipulation prior to the diagnosis of a spinal epidural hematoma [16]. 

This case is unique due to the lack of predisposing factors toward the development of a SSEH. With no history of hypertension or blood dyscrasias, and being of a young age, male sex is the only risk factor associated with SSEH in our patient. The most likely conclusion for the formation of the hematoma is stress toward the spine from weightlifting. A similar case of an exercise-induced epidural hematoma was in an elite-level swimmer. Other than an intense swim practice the day before, there was no traumatic event correlated with the development of a massive spontaneous epidural hematoma [17].

## Conclusions

SSEH presents with a variety of symptoms, potentially causing diagnostic delays, particularly in young patients with sudden neurologic deficits after activities such as weightlifting. It was concluded that the Valsalva maneuver while bench pressing put pressure toward the spine creating a hematoma, confirmed by hyperintensity on T2-weighted MRI. While SSEH commonly correlates with certain conditions, such as blood disorders, AV malformations, on platelet, thrombolytic, and/or anticoagulants, our patient lacked predisposing factors. Nonetheless, their prompt presentation, surgical intervention, and postoperative therapy likely contributed to a favorable outcome, upgrading their classification from AIS-A to AIS-C upon discharge, with retained rectal tone and sensation despite requiring assistance for ambulation.
